# The role of long non-coding RNAs in the development of adipose cells

**DOI:** 10.1016/j.ncrna.2023.02.009

**Published:** 2023-03-02

**Authors:** Albert Sufianov, Aferin Beilerli, Valentin Kudriashov, Tatiana Ilyasova, Yanchao Liang, Albert Mukhamedzyanov, Marina Bessonova, Andrey Mashkin, Ozal Beylerli

**Affiliations:** a Educational and Scientific Institute of Neurosurgery, Рeoples’ Friendship University of Russia (RUDN University), Moscow, Russia; b Department of Neurosurgery, Sechenov First Moscow State Medical University (Sechenov University), Moscow, Russia; c Department of Obstetrics and Gynecology, Tyumen State Medical University, 54 Odesskaya Street, 625023, Tyumen, Russia; d Gastric Cancer Center, West China Hospital of Sichuan University, China; e Department of Internal Diseases, Bashkir State Medical University, Ufa, Republic of Bashkortostan, 450008, Russia; f Department of Neurosurgery, The First Affiliated Hospital of Harbin Medical University, Harbin, 150001, China; g City Clinical Hospital №21, Ufa, Republic of Bashkortostan, 450071, Russia; h Tyumen Cardiology Research Center, Tomsk National Research Medical Center, Russian Academy of Science, Tomsk, Russia

**Keywords:** lncRNAs, Adipogenesis, Development, Energy metabolism, Obesity

## Abstract

In recent times, the rising prevalence of obesity and its associated comorbidities have had a severe impact on human health and social progress. Therefore, scientists are delving deeper into the pathogenesis of obesity, exploring the role of non-coding RNAs. Long non-coding RNAs (lncRNAs), once regarded as mere "noise" during genome transcription, have now been confirmed through numerous studies to regulate gene expression and contribute to the occurrence and progression of several human diseases. LncRNAs can interact with protein, DNA, and RNA, respectively, and participate in regulating gene expression by modulating the levels of visible modification, transcription, post-transcription, and biological environment. Increasingly, researchers have established the involvement of lncRNAs in regulating adipogenesis, development, and energy metabolism of adipose tissue (white and brown fat). In this article, we present a literature review of the role of lncRNAs in the development of adipose cells.

## 1 Introduction

Long non-coding RNAs (lncRNAs) was originally considered as a by-product of RNA polymerase II (Pol II) transcription, and was regarded as the "noise" of genome transcription because it cannot encode protein [[1], [2], [3], [4], [5]]. In recent years, with the help of high-throughput omics technology, more and more lncRNAs have been annotated. A large number of studies have shown that lncRNAs are widely involved in the regulation of gene expression, in cell proliferation, differentiation, apoptosis and tumor progression. important role in development [[1], [2], [3], [4], [5]]. The results of the latest research show that lncRNAs can still be translated into small molecular polypeptides, which extends the function of polypeptide-encoding genes in the human genome to a certain extent [6]. The etiology of obesity and the pathophysiological regulatory network have always been the focus of attention of scientists and the difficulty of research. More and more studies have proved that lncRNAs plays an important role in the occurrence and development of obesity [4,5].

## 2 LncRNAs Overview

### 2.1 Classification, characteristics and main functions of lncRNAs

**Fig. 1:**
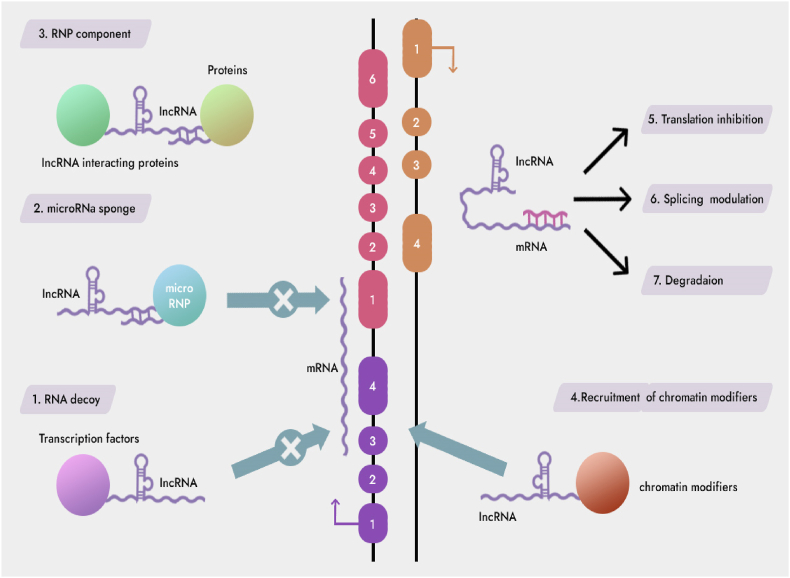
Mechanisms of lncRNAs functions.

Human genome sequencing shows that about 98% of RNAs do not have protein coding ability. These RNAs are collectively referred to as non-coding RNAs (ncRNAs), among which regulatory ncRNAs are one of them, mainly including short noncoding RNAs (small noncoding RNAs, sncRNAs) and lncRNAs [5,7]. Although the length of 200 nt can basically distinguish it from sncRNAs, it is not very accurate to define RNA with a length greater than 200 nt and no protein translation ability as lncRNAs, because some lncRNAs are less than 200 nt in length [1]. The latest study found that lncRNAs still has the ability to encode certain small molecule polypeptides, so the definition of lncRNAs is still open to question [6]. According to the location and background of lncRNAs in the genome, it can be divided into five types: intergenic, intronic, sense, antisense, and bidirectional lncRNAs (Fig. 1) [5,8].

LncRNAs structures are heterogeneous, with or without poly A tails [[1], [2], [3], [4]]. Compared with mRNA, the expression level of lncRNA is lower, and the conservation between species is poor, and its expression has obvious tissue or cell specificity, mainly localized in the nucleus, and a few localized in the cytoplasm [1,3,5]. Compared with miRNA, lncRNAs has a longer sequence, more complex spatial structure, and richer information content, so it may be a core of the genetic information expression regulatory network [9,10]. LncRNAs regulatory targets are related to factors such as their location in the genome, nucleotide sequence, and structure, and there is no universal mode of action, mainly through the RNA-RNA base pairing mechanism [5,11]. LncRNAs can interact with proteins, DNA, and RNA, and play different roles in various biological processes mainly through their functions as signals, baits, guides, and scaffolds [1,11,12].

### 2.2 LncRNAs regulation mode

At present, it is believed that lncRNAs mainly participates in the regulation of gene expression through the following levels.

#### 2.2.1 Regulation of epigenetic modification levels

LncRNA can participate in epigenetic regulation and gene expression through DNA methylation or demethylation, chromatin remodeling, histone modification, etc [13,14]. Specifically, lncRNAs can change chromatin structure; cause gene silencing through histone modification; bind to chromatin modification complexes and recruit related factors to specific sites; recruit chromatin modification inhibitors to participate in specific silencing alleles [13,14].

#### 2.2.2 Transcriptional regulation

LncRNAs can recruit transcriptional regulatory factors to adjacent target gene promoters, and regulate the expression of target genes through the cis mechanism or trans mechanism at the transcriptional level: activate the target gene by activating transcription factors, and enhance the transcription of the target gene [1]. It inhibits gene transcription by interfering with transcription, changing the subcellular localization of transcription factors, and competing with transcription factors for substrates [1,12,15].

#### 2.2.3 Post-transcriptional regulation

lncRNAs can form RNA dimers with target mRNAs, preventing mRNAs from binding to transcription or processing factors; lncRNAs can directly recruit translational repressor proteins to regulate splicing, translation, and mRNA degradation; lncRNAs can also participate in the splicing of mRNA precursors. mRNA capping, methylation and export to the cytoplasm. In addition, lncRNAs can also stabilize, stimulate, or inhibit translation of the target mRNA and even promote the degradation of the target mRNA [15,16].

#### 2.2.4 LncRNAs act as biological mediators due to their regulation

LncRNAs can act as a molecular scaffold to link two protein molecules together [[17], [18], [19], [20]]. It can localize a specific ribonucleoprotein complex in a specific part of the chromosome, thereby changing the expression of neighboring genes or distant genes in order to play its role as a molecular guide [18,20]. Some lncRNAs can act as molecular traps, inducing certain proteins, RNA or microRNA molecules (miRNAs) to leave certain areas [18,20]. They can compete with miRNA for mRNA and play the role of a “molecular sponge” [20,21]. LncRNAs can also act as regulators of specific signaling pathways [18,20].

## 3 LncRNAs and adipogenesis

The widespread prevalence of obesity has triggered scientists' research on the mechanism of action of lncRNAs on adipocytes. Studies in recent years found that the expression of adipose-derived lncRNA has obvious adipose tissue specificity [22]. The expression of sexual lncRNAs is low and has obvious anatomical location specificity. The expression of lncRNAs in abdominal fat and buttock fat in obese people is also different [22]. With the help of high-throughput sequencing or microarray technology, more and more lncRNAs have been confirmed to be involved in the adipogenic and development of white fat and brown fat (Table 1).

**Table 1:** Adipogenesis related long noncoding RNAs.

lncRNA	Features, categories	Cells, tissues	Function in adipogenesis	References
**White adipose tissue**				
*ADNCR*	Poly A-tailed, intergenic lncRNA	preadipocyte	Inhibiting adipogenic differentiation	[21]
*Lnc-RAP-1*	Poly A-tailed, intergenic lncRNA	mouse primary adipocytes	Promoting adipogenic differentiation	[25]
*SRA*	Poly A-tailed, intergenic lncRNA	3T3-L1 cells, ST2 cells, liver	Promoting adipogenesis and inhibit the expression of ATGL	[28,[31], [32], [33]]
*NEAT1*	mainly expressed in the nucleus	Adipose-derived mesenchymal stem cells	Promoting adipogenic differentiation	[34]
*ADINR*	Poly A-tailed, bidirectional lncRNA, mainly expressed in the nucleus	human bone marrow mesenchymal stem cells	Promoting adipogenic differentiation	[35]
*HoxA-AS3*	antisense lncRNA	mesenchymal stem cells	Promoting directed adipogenesis of bone marrow mesenchymal cells	[36]
*PU.1 AS lncRNA*	antisense lncRNA	3T3-L1 cells, porcine primary adipocytes	Promoting adipogenic differentiation	[38]
*HOTAIR*	HOX antisense intergenic RNA	human adipose-derived mesenchymal stem cells	Participating in metabolism; promote adipogenic differentiation	[22,39,40]
*SlincRAD*	Non-poly A-tailed, intergenic lncRNA	preadipocyte	Promoting adipogenic differentiation	[41]
*LncRNA-H19*		bone marrow mesenchymal stem cells	Inhibiting adipogenic differentiation	[43]
*LncRNA U90926*	expressed in the cytoplasm	3T3-L1 cells	Inhibiting adipogenic differentiation	[44]
**Brown adipose tissue**				
*Blnc1*	Poly A-tailed, intergenic lncRNA, mainly expressed in the nucleus	Mouse primary brown adipocytes	Promotes brown adipogenicity and stimulates thermogenic gene expression	[48]
*Lnc-BATE1*	Poly A-tailed, intergenic lncRNA, Expressed in the cytoplasm and nucleus	Mouse primary brown adipose tissue	Maintains brown fat content and thermogenic function	[49]
*uc.417*	Poly A-tailed, intergenic lncRNA,	Mouse primary brown adipocytes	Promotes adipogenic differentiation of brown fat	[50]
*GM13133*	antisense lncRNA	brown fat cells	Inhibits the differentiation of white adipocytes and promote the browning of white adipocytes	[52]
*LncBATE10*	Expressed both in the nucleus and in the cytoplasm, intergenic lncRNA	brown fat cells	Promotes adipogenic differentiation of brown fat and browning of white adipose tissue	[53]

**Abbreviations:** SRA: steroid receptor RNA activator; NEAT1: nuclear-rich transcription factor 1; ADINR: adipogenic differentiation-inducing non-coding RNA; HOTAIR: HOX transcript antisense RNA; ADNCR: adipocyte differentiation-associated long non-coding RNA; Blnc1: brown fat lncRNA1; ATGL: adipose triacylglycerol lipase.

### 3.1 LncRNAs and adipogenicity and development of white fat

Luan et al. obtained a transcriptome profile of human adipose tissue-derived mesenchymal stem cells (*hADSC*) during adipogenic differentiation and identified 2868 differential mRNAs and 207 ncRNAs [23]. Cooper et al. used high-throughput sequencing technology to analyze the expression profiles of brown fat and white fat in mice at different developmental stages and found that compared with mature adipocytes, preadipocytes had more than 100 lncRNAs differentially expressed, and in preadipocytes. The promoter regions of some lncRNA genes highly expressed in cells have binding sites for the key transcription factor peroxisome proliferator receptor gamma (*PPARγ*) in adipocyte differentiation [24]. Interfering with these lncRNAs can lead to the obstruction of preadipocyte differentiation. Sun and colleagues analyzed the expression profiles of mRNA and poly-A tail lncRNAs during adipocyte differentiation and found that 1734 coding genes and 175 lncRNAs were strongly induced to be expressed during white and brown adipocyte differentiation [25]. LncRNAs were upregulated during brown adipocyte and white adipocyte differentiation, and 10 lncRNAs with the strongest regulatory effect were knocked out. After a single injection of lncRNA, mature adipocytes can almost completely dedifferentiate into preadipocytes, and lipid accumulation in adipocytes is significantly reduced. In addition, a group of lncRNAs associated with fat metabolism or adipocyte differentiation has also been studied in humans, mice, cattle, pigs and other species that play an important regulatory role in differentiation [[26], [27], [28], [29], [30]].

#### 3.1.1 LncRNAs that promote adipogenesis and development of white fat

Steroid receptor RNA activator (SRA) is the first class of lncRNA found to be involved in adipogenesis [31]. SRA in adipose tissue enhances its transcriptional activity by binding to PPARγ, thereby promoting the differentiation of 3T3-L1 preadipocytes; SRA can regulate the cell cycle of adipocytes, insulin-related signal transduction pathways, and TNF-α signaling pathways [31]. The expression of SRA in the white adipose tissue of obese mice induced by high-fat diet was significantly increased, while the fat content of SRA knockout mice was decreased. The expression of a large number of adipocyte markers and inflammatory genes is reduced, insulin sensitivity is enhanced, and it can resist obesity and fatty liver induced by high-fat diet [28]. SRA in adipocytes increases glucose uptake, leading to phosphorylation of serine-threonine protein kinase (AKT) and forkhead box transcription factor 1 (FoxO1) in the insulin signaling pathway; prolongs the S phase of the cell cycle; reduces cyclins Rely on enzyme inhibitors *p21Cip1* and *p27Kip1*, increase phosphorylation of cyclin-dependent kinases *Cdk1/Cdc2;* inhibit adipocyte-associated inflammatory gene expression and TNF-α-induced phosphorylation of *c-JUN-NH(2)* terminal kinase [31]; SRA overexpression can inhibit the phosphorylation of *p38MAPK* and JNK kinases during early differentiation of ST2 cells, while knocking out SRA in 3T3-L1 cells can lead to a decrease in insulin receptor levels and a weakened insulin receptor β (IR-β) autophosphorylation, decreased phosphorylation of insulin receptor substrate 1 and AKT. SRA also increases the transcription of the insulin receptor [32]. SRA in the liver can also block the promoter activity of adipose triglyceride lipase (ATGL) by inhibiting the related effects of transcription factor FoxO1, thereby inhibiting the expression of ATGL to promote liver steatosis and participate in the body's fat metabolism [33].

Nuclear-enriched transcription factor 1 (NEAT1) is a class of nuclear lncRNAs closely related to the paraspeckle form [24]. NEAT1 has a binding site for miR-140, and the mature miR-140 in the nucleus can physiologically interact with NEAT1, leading to an increase in the expression of the latter. Gernapudi et al. found that *miR-140* can physiologically bind to NEAT1 and increase its expression [34]. In primary mouse adipose-derived stem cells (ADSCs), the knockout of *miR-140* led to down-regulation of NEAT1 expression, the expression of adipocyte markers was reduced, and overexpression of NEAT1 in miR-140-knockout ADSCs could promote adipocyte differentiation again, thereby restoring the adipogenic phenotype, indicating that NEAT1 can participate in miR-140-induced adipogenicity.

Adipogenic differentiation-inducing non-coding RNA (ADINR) is an lncRNA located 450 bp upstream of the CCAAT/enhancer binding protein α (CCAAT/enhancer binding protein α, C/EBPα) gene. Its expression is significantly up-regulated during the adipogenic differentiation of human bone marrow mesenchymal stem cells (MSCs). Knockdown of ADINR affects adipogenesis, whereas overexpression of C/EBPa can restore impaired adipogenesis due to loss of endogenous ADINR [35]. ADINR can specifically bind to the subunit PA1 of *MLL3/4* to form the *MLL3/4* histone methyltransferase complex, thereby increasing the trimethylation (H3K4me3) of histone 3 subunit lysine 4 in C/EBα The modification of the gene weakens the trimethylation (*H3K27me3)* of the 3rd subunit of histone lysine 27 (*H3K27me3*) in the modification of the C/EBPa gene, and finally promotes the activation of C/EBPa and enhances adipogenesis. Therefore, ADINR can regulate the target gene C/EBPa through a *cis*-action mechanism and regulate the adipogenic differentiation of MSCs [35].

LncRNAs can regulate the reprogramming of gene expression profiles during MSCs commitment and maturation [36]. The expression of HoxA-AS3 was significantly up-regulated in MSCs induced by adipogenic orientation, but not changed in osteogenic induction; silencing HoxA-AS3 could inhibit MSCs adipogenic and promote osteogenic differentiation. Studies found that HoxA-AS3 can bind to the enhancer of histone methyltransferase (EZH2), and HoxA-AS3 is required for the H3K27me3 modification of the key osteogenic transcription factor-Rount-related transcription factor 2 (Runx2) [36]. Therefore, HoxA-AS3 may be an important epigenetic switch that determines the lineage-specific differentiation of MSCs.

AS lncRNAs are a class of lncRNAs transcribed from opposite DNA strands and partially overlapping sense mRNAs. PU.1 is a transcription factor that can inhibit the differentiation of preadipocytes [37]. PU.1 AS lncRNA can combine with PU.1 mRNA in preadipocytes to form mRNA/AS lncRNA diploid, preventing PU.1 mRNA from translation, thereby promoting adipogenesis [38]. After knocking out PU.1 AS lncRNA in 3T3-L1 cells, the binding of PU.1 AS lncRNA to PU.1 was reduced to promote the expression of PU.1 protein, resulting in reduced lipid accumulation and adipocyte markers (such as *PPARγ, C/EBPa*) expression decreased and inhibited adipogenesis [38].

HOX Transcript Antisense Strand RNA (HOTAIR) is an antisense HOX intergenic RNA localized at the HOXC locus that is involved in metabolic processes and differentiation of human subcutaneous adipose tissue preadipocytes [22,39,40]. HOTAIR is expressed in human gluteal muscle fat but not in abdominal subcutaneous fat, but its expression is increased during preadipocyte differentiation. When HOTAIR is ectopically expressed in abdominal preadipocytes, it can increase the proportion of adipocytes and increase the expression of adipogenic markers without affecting the preadipocyte proliferation coefficient, indicating that this lncRNA is involved in adipocyte differentiation from preadipocytes to mature adipocytes and the lipid process. the process of droplet accumulation and has a site specificity [22]. In addition, very long chain intergenic transcripts also have an important influence on adipogenesis. Yi et al. found that a class of extra-long intergenic non-coding RNAs (slincRAD) with a length of about 136 kb. is involved in adipose differentiation, and suppression of slincRAD in preadipocytes by specific siRNAs can lead to lipid differentiation during adipose tissue differentiation [41]. Accumulation is reduced and PPARγ levels are reduced. LncRAP-1, also known as FIRRE (Functional Intergenic Repeat RNA Element) or 6720401G13Rik, is an intergenic lncRNA located on the X chromosome, spanning a 5 Mb domain spatially adjacent to the nucleus 5 of the locus of various trans chromosomes, which can interact with nuclear structure of the chromosome to play a regulatory role [25,42]. During adipogenic differentiation, lnc-RAP-1 can bind heterogeneous nuclear ribonucleoprotein U (hnRNP-U), required for adipogenicity, to regulate the expression of adipogenic factors [25,42].

#### 3.1.2 LncRNAs that inhibits adipogenesis and development of white fat

*LncRNA-H19* is a newly discovered lncRNA that can inhibit the adipogenic differentiation of BMSCs, and it can exert its inhibitory effect through the epigenetic modification of histone deacetylase [43]. *LncRNA-h19* can work synergistically with miR-675: miR-675 can degrade adipogenic histone deacetylase 4–6 (HDAC4-6). As an HDAC inhibitor, trichostatin can significantly reduce the presence of CCCTC binding factor (CCCTC binding factor, CTCF) in the upstream imprinting control region of the H19 gene locus, down-regulate the expression of lncRNA-H19, and play an important role in the directed adipogenesis of BMSCs.

*LncRNA* U90926 is also a type of adipose tissue-specific lncRNA, its expression in obese mice is significantly lower than that in non-obese mice, its expression decreases with the differentiation of 3T3-L1 preadipocytes, and its overexpression can attenuate 3T3-L1 adipogenic differentiation. Knockout has the opposite effect, *lncRNA* U90926 can attenuate the adipogenic differentiation of 3T3-L1 cells by inhibiting the transactivation of PPARγ or PPARγ2 [44].

Adipocyte differentiation-associated long non-coding RNA (ADNCR) is a newly discovered lncRNA whose expression is significantly down-regulated during adipogenic differentiation of calf adipocytes [21]. As a miR-204-competing endogenous RNA (ceRNA), ADNCR can competitively increase the expression of the target gene silent information regulator 1 (SIRT1) in a miR-204-dependent manner and inhibit adipocyte differentiation.

### 3.2 LncRNAs and brown fat adipogenic development, white fat beige and energy metabolism

Brown fat and beige fat in the white fat area not only have the function of thermogenesis, but also regulate other metabolic tissues to affect the energy metabolism of the system [45]. The role will further expand the idea of obesity diagnosis and treatment. Brown adipocytes and muscle cells have a very similar transcription process, and lncRNA is involved in the differentiation of brown adipocytes and muscle cells [46]. Brown adipocytes and myocyte-specific lncRNAs have differential effects on transcription by regulating their transcriptional targets during molecular formation, and lncRNAs are involved in controlling energy metabolism in brown adipose tissue and other energy-consuming tissues, and promoting adipose tissue consumption [47,48].

Brown fat lncRNA1 (Blnc1), as a type of nuclear lncRNAs, is specifically expressed in brown fat and can form a ribonucleoprotein complex with the transcription factor Early B cell factor 2 (EBF2) to stimulate the production of Thermogene expression, and also promote differentiation of brown and beige EBF2 target adipocytes also stimulates adipogenicity in cells with a thermogenic phenotype [49]. Lnc-BATE1 is an important regulator of brown adipose tissue development and physiological functions and is required to maintain brown fat content and thermogenesis [50]. Lnc-BATE1 can function by binding to hnRNP-U, and both lnc-BATE1 and hnRNP-U are required for brown fat development [50]. In addition, there is a mechanistic link between zinc finger and BTB domain 7b (Zbtb7b), which is required for the activation of thermogenic genes in brown adipocytes and beige adipocytes and lncRNAs regulatory pathways, and Zbtb7b can recruit the Blnc1/hnRNPU complex [45].

Uc.417 is an lncRNAs transcribed from an ultra-conserved region found in rodents, and its expression gradually increases during the adipogenic differentiation of brown fat. Although uc.417 is not required for the function of brown adipose tissue, it can inhibit the thermogenesis induced by cold stimulation in mouse brown adipose tissue, and its ectopic expression can impair the adipogenic and thermogenic functions of brown adipose tissue. The p38MAPK signaling pathway is critical for activated brown fat-induced uncoupling protein-1 (UCP-1) expression, and uc.417 mildly inhibits p38MAPK without affecting total p38MAPK protein levels Phosphorylation of brown adipocytes regulates the adipogenic differentiation process of brown adipocytes by mediating DNA methylation, histone modification and other epigenetic methods [51].

GM13133 is a brown fat-specific lncRNAs that is regulated by cold and β3-adrenergic agonists and cyclic adenosine monophosphate stimulation, and has a potential role in the beigeization of white fat [52]. Although GM13133 overexpression does not affect the proliferation of white preadipocytes in mice, it can make white adipocytes beige by increasing mitochondrial activity and inducing the expression of brown fat-specific markers. This process may be related to the regulation of cAMP signaling pathway [52].

A recent study showed that lncBATE10 is a type of lncRNA highly expressed in brown adipose tissue, which is controlled by the cAMP-cAMP binding protein (CREB) axis and is required for complete differentiation of brown adipose tissue, and the beige coloration of white adipose tissue is sparse [53]. Mechanically, lncBATE10 can divert the CUGBP Elav-like family member 1 (Celf) from a specific region of the peroxisome proliferator-activated receptor gamma-coactivator 1α (PGC-1α), thereby interfering with the Celf1 response to PGC-1α inhibition [53].

Recent studies have established that several lncRNAs not only influence adipogenesis and development of brown adipose tissue, but also play an important role in the differentiation and maturation of white adipose tissue or muscle tissue [54,55]. For example, AK142386 and AK133540 may be involved in the development of brown fat and white fat through the corresponding target genes *HOXA3* and *Acad10*, while *lncRNA* AK003288 may be involved in the differentiation, maturation, and interaction of brown fat and skeletal muscle. affecting affinity protein 2 (Jph2) [54,55].

In addition to adipose tissue, lncRNAs in other key metabolic organs have been confirmed to be involved in the regulation of energy metabolism [56]. Yang et al. annotated a class of lncRNAs as important regulators of metabolism in key metabolic organs in the typical metabolic state of mice and established a systemic framework for studying the role of lncRNAs in physiological metabolic homeostasis [56]. At the same time, it was confirmed in primary hepatocytes that liver metabolism-sensitive lncRNA (*lncLMS*) is specifically regulated by nutrients, metabolic hormones, and key transcription factors; the metabolic regulation of lncRNAs was confirmed, and it was found that lncRNAs in mice can bind to the sterol regulatory element binding protein -1c (*SREBP-1c*) and can form a negative feedback loop to inhibit adipogenesis.

In conclusion, with the deepening of research on lncRNAs in the field of metabolic diseases, the metabolic network diagram centered on energy metabolism is becoming clearer and clearer, which provides a basis for further elucidation of the transcriptomic changes accompanying energy homeostasis transition in adipose tissue [53].

### 3.3 LncRNAs and obesity and metabolic diseases

With the deepening of basic research on obesity and related metabolic diseases, clinical research involving lncRNAs and obesity and metabolic diseases has also begun to increase. However, current research at the clinical level mainly focuses on the clinical value of lncRNAs as a novel biomarker related to obesity and related metabolic diseases. For example, the development of new biomarkers for obesity diagnosis and treatment, the development of new drug targets for obesity treatment, and lncRNAs does have the potential to become a biomarker in fat metabolism-related diseases [3]. Ballantyne et al. found that intergenic lncRNAs (lincRNAs) were associated with metabolic diseases through whole genome sequencing and trajectory tracking, among which *linc-NFE2L3-1* expressed in human adipose tissue was significantly correlated with waist-to-hip ratio after correcting BMI [57]. Sun et al. found that the lncRNAs in the blood was significantly abnormal in the obese population through genome-wide lncRNAs sequencing research on the blood of obese and non-obese people [58]. Circulating levels of 3 lncRNAs (*lncRNA-p5549, lncRNA-p21015, lncRNA-p19461*) were specifically decreased in obese people and negatively correlated with waist circumference, waist-to-hip ratio, fasting insulin and BMI levels. At the same time, the expression of *lncRNA-p19461* was negatively correlated with the homeostasis model of insulin resistance (HOMA-IR); in addition, the level of *lncRNA-p19461* was also significantly increased in the blood of 8 obese patients who dieted for 12 weeks. The lncRNA in the liver may also participate in the fat metabolism of the body and play a role in the formation of hepatic steatosis or liver fibrosis, such as SRA promoting hepatic steatosis; lung adenocarcinoma metastasis-associated transcription factor 1 (*MALAT1*) is involved in nonalcoholic Inflammation and fibrosis in liver disease [59,60].

LncRNAs shown to be associated with diabetes. In a recent study by Sanchez-Parra et al., it was found that H19 may regulate miRNA let-7 and the activation of Akt to promote β-cell expansion in newborns and adult β-cell proliferation, and its silencing decreased β-cell expansion [61]. H19 has also been shown to have potential as a biomarker in insulin resistance, as circulating levels have been found to be significantly increased in T2D patient cohorts [62].

MEG3, another well-known lncRNAs, is mostly indicated in cancer suppression, but its expression has been associated with pancreatic cell apoptosis, insulin synthesis, and secretion, and its overexpression may promote hepatic insulin resistance by acting as a sponge for miR-214 and increasing FOXO1 expression [63]. Furthermore, MEG3's competitive binding to miR-185-5p as a competing endogenous RNA (ceRNA) has been shown to promote the expression of early growth response 2 (EGR2), which inhibits IRS, and its overexpression in T2D patients has been significant [64,65].

Like other lncRNAs, MALAT1 is also associated with oxidative stress-induced insulin resistance, as it suppresses insulin signaling by inhibiting the phosphorylation of IRS and Akt through the upregulation of the stress-sensitive kinase c-Jun N-terminal kinase (Jnk), which has been shown to negatively regulate insulin signaling. MALAT1 expression has been found to be increased in GDM patients, further supporting its negative regulatory effect [66,67].

LncRNAs shown to be associated with elevated blood pressure. During a discovery phase involving RNA-sequencing and a validation phase using quantitative polymerase chain reaction, two lncRNAs (TCONS_00028980 and TCONS_00029009) were found to have differential expression between Dahl salt-sensitive rats and salt-insensitive, congenic Brown Norway SS.13 rats exposed to a high-salt diet, indicating a potential role in hypertension [68]. Genetic research in humans also supports the role of specific polymorphisms within the lncRNA CDKN2B-AS1 (rs10757274, rs2383207, rs10757278, and rs1333049) in increasing susceptibility to developing essential hypertension [69]. Furthermore, a microarray analysis of renal cortex tissue revealed 145 differentially expressed lncRNAs between spontaneously hypertensive rats and normotensive Wistar-Kyoto rats, further suggesting a potential involvement of lncRNAs in the pathogenesis of hypertension [70]. In addition, four lncRNAs (TCONS_00052110, TCONS_00201718, TCONS_00094247, and TCONS_00296056) were found to be upregulated in failing right ventricles of Sprague-Dawley rats treated with monocrotaline to establish pulmonary arterial hypertension and lipopolysaccharide to induce acute inflammation and heart failure [71]. MANTIS, a lncRNA related to the angiogenic function of endothelial cells, was found to be downregulated in patients with pulmonary arterial hypertension (PAHT) and rats treated with monocrotaline. Inhibition of MANTIS through various methods had positive effects on endothelial cells under shear stress, indicating its potential as a therapeutic option for hypertension [72]. Another lncRNA, Giver (growth factor- and proinflammatory cytokine-induced vascular cell-expressed RNA), was found to be upregulated in arteries from hypertensive patients and involved in Ang II-mediated vascular smooth muscle cell dysfunction. Its downregulation after treatment with angiotensin-converting enzyme inhibitors and angiotensin receptor blockers suggests its potential as an antihypertensive drug [73]. The lncRNA H19 was found to be upregulated in serum and lung samples from rats and mice after monocrotaline treatment, and this was associated with proliferation of pulmonary arterial smooth muscle cells [74]. The upregulation of lncRNA-AK098656 was observed in the plasma of individuals with hypertension, and it was found to enhance the proliferation of VSMCs. Additionally, the transgenic rats that overexpressed lncRNA-AK098656 showed a spontaneous development of hypertension along with narrowed resistant arteries [75].

## 4 Conclusion

New ideas and approaches for preventing and treating obesity have emerged from research into the role and mechanism of lncRNAs in adipogenesis, development, and energy metabolism [[76], [77], [78], [79]]. Recent research has identified lncRNAs as gene carriers for patrilineal inheritance of obesity and their participation in obesity-induced infertility [80]. The involvement of lncRNAs in various metabolic diseases and related complications is becoming increasingly apparent. However, other factors, such as lipolysis and mitochondria, also contribute to the pathogenesis of obesity. Although research on β-oxidation and other aspects rarely involve lncRNAs, external nutrition, signals, and stress states have a significant influence on adipose tissue due to its interaction network with other organs or tissues, including the central nervous system, important metabolic organs, and the immune system. The dynamic changes of cell lipid droplets and the regulation of lipid metabolism are scientific issues worthy of further exploration. The formation and metabolism of lipid droplets play an essential role in the growth and development of fat, and lipid droplets in adipocytes and other parts, such as muscle and ectopic deposition in the liver, play a vital role in the pathophysiological mechanism of obesity [4]. LncRNAs may participate in regulating related metabolic diseases through lipid droplets as carriers, specifically by expressing small peptides that affect lipid droplet formation. LncRNAs can also interact with miRNAs to affect lipid droplet binding proteins and/or other regulators of lipid droplet formation [81,82]. Recent studies have shown that lncRNAs are also involved in regulating the body's immune system [83]. Some natural antisense lncRNAs can even be used as therapeutic targets for human immune diseases. As obesity is an atypical immune disease, lncRNAs can also affect its development. In the future, the field of lncRNAs will increasingly involve various aspects of the pathogenesis of obesity and related networks. The pathophysiological network centered on obesity will become clearer, and more drug targets for obesity will emerge.

## Funding

This work was supported by the Bashkir State Medical University Strategic Academic Leadership Program (PRIORITY-2030).

## Author contributions

Albert Sufianov and Aferin Beilerli conceptualized and designed the study. All authors have participated in the acquisition, analysis and interpretation of the data. Marina Bessonova, Andrey Mashkin and Valentin Kudriashov has drafted the manuscript. Tatiana Ilyasova, Yanchao Liang and Albert Mukhamedzyanov contributed to the critical revisions of the manuscript. Ozal Beylerli supervised the research. All authors agreed on the journal to which the article would be submitted, gave the final approval for the version to be published, and agreed to be accountable for all aspects of the work.

## Declaration of competing interest

The authors declare that no conflicts of interest exist.
